# Advanced technologies for reducing greenhouse gas emissions from rice fields: Is hybrid rice the game changer?

**DOI:** 10.1016/j.xplc.2024.101224

**Published:** 2024-12-28

**Authors:** Seyed Mahdi Hosseiniyan Khatibi, Maria Arlene Adviento-Borbe, Niña Gracel Dimaano, Ando M. Radanielson, Jauhar Ali

**Affiliations:** 1 International Rice Research Institute, Metro Manila, Philippines; 2 US Department of Agriculture (USDA) Agricultural Research Service (ARS), USA; 3 College of Agriculture and Food Science, University of the Philippines Los Baños, Laguna, Philippines

**Keywords:** hybrid rice, climate change, machine learning, nitrous oxide, N_2_O, methane, CH_4_, greenhouse gas

## Abstract

Rice is a staple food for half of the world’s population and the largest source of greenhouse gas (GHG) from the agricultural sector, responsible for approximately 48% of GHG emissions from croplands. With the rapid growth of the human population, the increasing pressure on rice systems for extensive and intensive farming is associated with an increase in GHG emissions that is impeding global efforts to mitigate climate change. The complex rice environment, with its genotypic variability among rice cultivars, as well as emerging farming practices and global climatic changes, are important challenges for research and development initiatives that aim to lower GHG emissions and increase crop productivity. A combination of approaches will likely be needed to effectively improve the resilience of modern rice farming. These will include a better understanding of the major drivers of emissions, different cropping practices to control the magnitude of emissions, and high yield performance through systems-level studies. The use of rice hybrids may give farmers an additive advantage, as hybrids may be better able to resist environmental stress than inbred varieties. Recent progress in the development and dissemination of hybrid rice has demonstrated a shift in the carbon footprint of rice production and is likely to lead the way in transforming rice systems to reduce GHG emissions. The application of innovative technologies such as high-throughput sequencing, gene editing, and AI can accelerate our understanding of the underlying mechanisms and critical drivers of GHG emissions from rice fields. We highlight advanced practical approaches to rice breeding and production that can support the increasing contribution of hybrid rice to global food and nutritional security while ensuring a sustainable and healthy planet.

## Teaser

Rice contributes to 48% of GHG emissions from croplands. This review discusses opportunities for lowering GHG emissions and increasing crop productivity through the use of hybrid rice. The adoption of high-yielding, shorter-duration rice hybrids that have been identified as low GHG emitters could help to significantly reduce GHG emissions. Data-driven approaches for identifying ways to reduce GHG emissions from rice paddies are highlighted.

## Introduction

**Figure 1:**
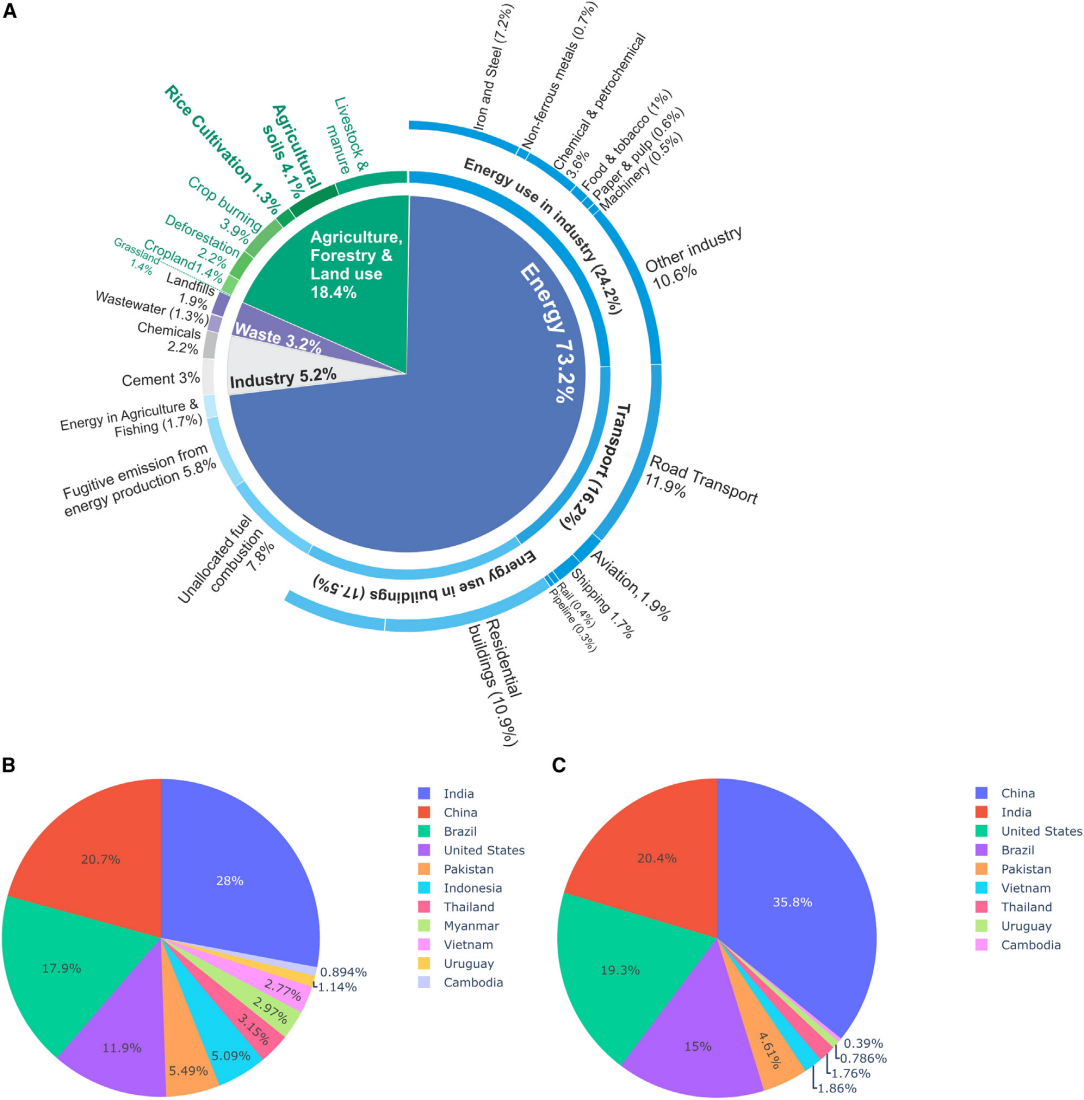
Contributions of the agricultural sector and leading rice-producing countries to global GHG emissions. **(A)** Right: GHG emissions (CO_2_, CH_4_, and N_2_O converted to CO_2_ equiv using a 100-year time horizon) from different segments of the agricultural sector as of 2020. This is the latest breakdown of global emissions by sector published by Climate Watch and the World Resources Institute (Pachauri et al., 2014). **(B and C)** Percentages of CH_4_ and N_2_O emissions contributed by the top 10 rice-producing countries (Ritchie, 2020; Ritchie and Roser, 2023).

The most critical problem facing humanity in this century is climate change, brought about by rising atmospheric greenhouse gas (GHG) emissions. A remarkable proportion of worldwide anthropogenic methane (CH_4_) and nitrous oxide (N_2_O) emissions comes from agriculture (Esmizade et al., 2010; Mikhaylov et al., 2020; Slingo and Slingo, 2024). Agricultural operations produce the majority of non-carbon dioxide (non-CO_2_) emissions (84% N_2_O and 47% CH_4_), which also account for 10%–17% of all human GHG emissions (Beach et al., 2015; Bellarby et al., 2008; Frank et al., 2019; Linquist et al., 2018; Springmann and Freund, 2022). Agricultural soils alone account for 4.1% of total GHG emissions, and rice cultivation is responsible for 1.3% (Ritchie, 2020), as shown in Figure 1A. Rice (*Oryza sativa* L.) is an essential food crop and the second most widely cultivated cereal crop worldwide (Bodie et al., 2019). In Asia, rice is a critical and nutrient-dense staple food. Although China and India account for the majority of rice consumption, overall consumption has grown significantly, rising from 157 million tons in 1960 to 520 million tons in 2022 (USDA, 2023). By 2030, consumption is predicted to increase by an additional ∼6% (Bin Rahman and Zhang, 2023). To meet the rising demand from the accelerated increase in the human population, rice output must be raised by 40% by 2030, which may cause significant environmental problems (IMF and UNCTAD, 2011). As a result, rice crop systems will need to be balanced by producing higher grain output with potentially lower GHG emissions.

Rice fields cover about 1.7 million km^2^ (Liu et al., 2021b) in 114 nations, accounting for 11% of all arable land worldwide (Gupta et al., 2021). It is estimated that approximately 11% and 30% of global agricultural N_2_O and CH_4_ are emitted from rice fields, respectively (Hussain et al., 2015; Ritchie and Roser, 2024). Figure 1B and 1C present the contributions of CH_4_ and N_2_O emissions from leading rice-producing countries to global GHG emissions. The rice-crop global warming potential (GWP) is 467% and 169% higher than those of wheat and maize (Linquist et al., 2012). Anaerobic soil conditions are conducive to CH_4_ formation, whereas N_2_O is produced mainly under aerobic conditions. The soil microbial processes of nitrification and denitrification create this N_2_O gas (Islam et al., 2020). Maximum amounts of CH_4_ are released when rice fields are continuously flooded, whereas significant amounts of N_2_O are generated under a dry cycle when rice is intermittently flooded and crops are rotated (Zhao et al., 2011). According to predictions, emissions of both GHGs could rise by 35%–60% by 2030 (Netz et al., 2007).

To establish mitigation methods and reduce the harmful effects of future climate crises, we must improve our understanding of the mechanisms of GHG emissions from rice fields and re-conceptualize the complex field environment of current and future rice production systems. In this review, we clarify the processes of GHG emissions, the primary drivers of emissions, and the potential for rice-based farming to reduce GHG emissions through climate-smart crop management systems. The wide-scale adoption of rice hybrids with a low CH_4_ footprint could be a game changer, together with a number of cutting-edge approaches to decrease future GHG emissions from rice fields.

## Mechanisms of CH_4_ emission from rice fields

CH_4_ emission is one of the main components of anthropogenic GHG emissions from rice fields (Ciais et al., 2014; Tian et al., 2016). CH_4_ is produced through the microbial process of methanogenesis, which requires anoxic conditions and a low redox potential (*E_h;_* < −150 mV). In rice soils, members of the domain Archaea facilitate CH_4_ production using fermentation products, i.e., alcohols, acetate, CO_2_, and H_2_ generated by other microorganisms during decomposition of plant matter and root exudates. Methanogenesis occurs by three biochemical pathways catalyzed by the enzyme methyl reductase. The hydrogenotrophic pathway involves the reduction of H_2_ to CO_2_ and produces CH_4_. The acetoclastic pathway entails splitting acetate, oxidizing the carbonyl portion of the organic molecule to CO_2_, and reducing the methyl portion to CH_4_. Methylotrophic pathways involve the production of CH_4_ from the methyl portion of organic compounds like methanol, methylamines, and dimethyl sulfide (Conrad, 2007; Conrad et al., 2007). Only one-third of methanogenesis in rice fields is derived from hydrogen with CO_2_ reduction; the rest is derived mainly from acetate (Conrad, 2007). Rice plants influence CH_4_ emissions by supplying root C substrates to methanogens, with the resulting CH_4_ carried to the atmosphere through root aerenchyma (Win et al., 2012; Kim et al., 2018). It has been reported that 90%–95% of total seasonal CH_4_ emissions exit the soil through rice plants and that 5%–10% of total seasonal CH_4_ emissions come from ebullition (Aulakh et al., 2000; Adviento-Borbe et al., 2015; Komiya et al., 2015).

**Figure 2:**
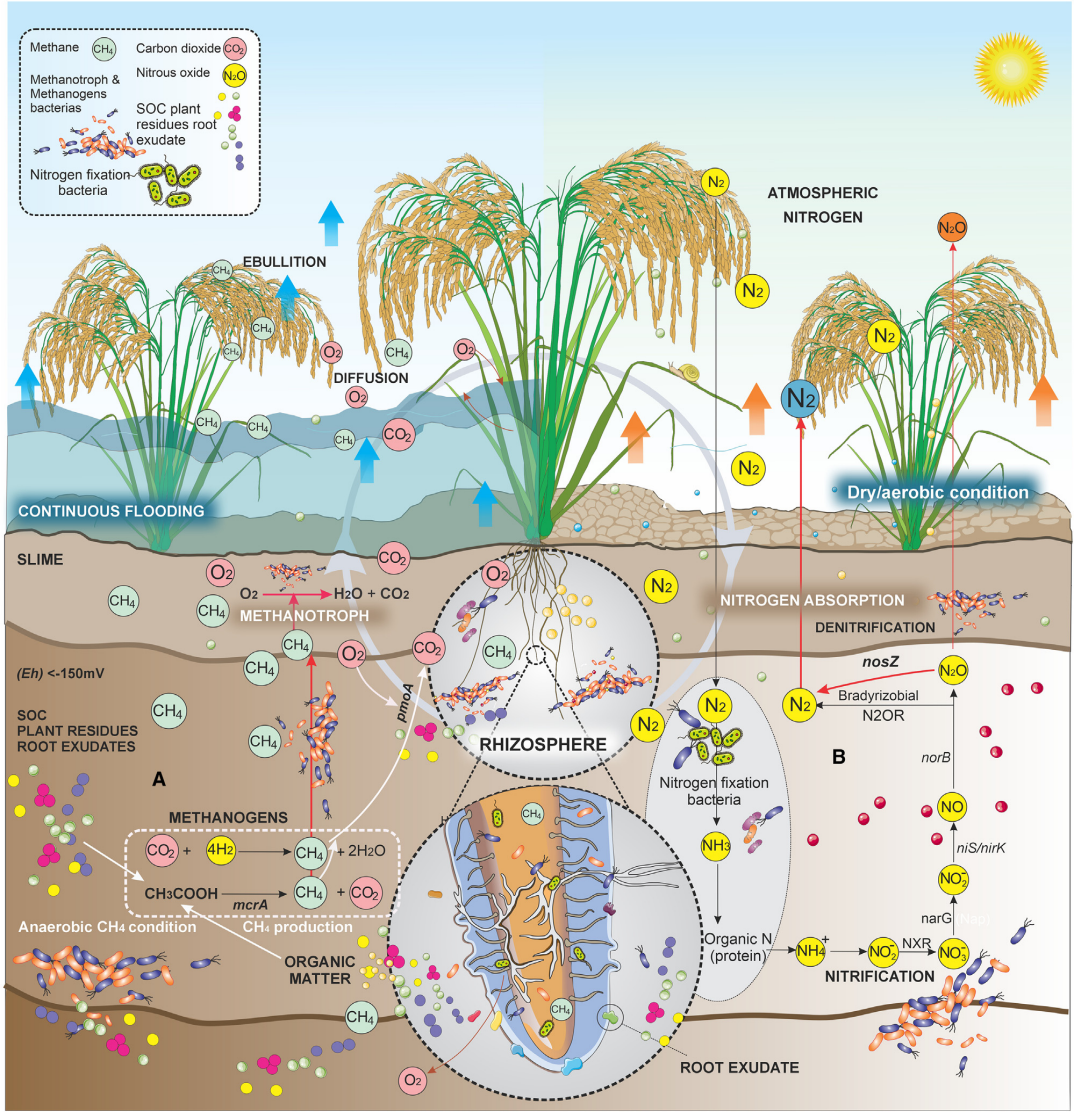
CH_4_ and N_2O_ production in the rice field. The illustration depicts the complex interactions among rice roots, soil environments, and microbial communities, highlighting their roles in the production and oxidation of **(A)** methane (CH_4)_ and **(B)** nitrous oxide (N_2_O). Rice root exudates influence microbial activity in the rhizosphere, promoting both the generation of N_2_O through nitrification and denitrification processes and the oxidation of CH_4_ by methanotrophic bacteria. The dynamic relationship between rice roots and microbial communities is influenced by soil properties and water management practices, which regulate the balance of GHG emissions.

Aerobic methanotrophs in the upper soil layer and rhizosphere, where O_2_ and CH_4_ gradients coincide, can convert the CH_4_ generated in the anoxic soil layer of rice fields to CO_2_ through a process known as methanotrophy or CH_4_ oxidation. Methanotrophs regulate the amount of CH_4_ gas that reaches the atmosphere. Previous studies have estimated that CH_4_ emissions from paddy rice could be 10%–60% higher without aerobic methanotrophs. Studies have shown that an increase in tiller number (Dubey and Singh, 2000) and plant biomass (Eller and Frenzel, 2001) can enrich the activity of CH_4_-oxidizing microbes by enhancing O_2_ transport and enlarging the volume of aerenchyma cells. Once CH_4_ has been produced, it is released into the atmosphere by several pathways: (i) diffusion loss of dissolved CH_4_ across the water–air and soil–water interfaces, (ii) ebullition loss by the release of gas bubbles, and (iii) plant transport into the roots by diffusion of CH_4_ gas in the aerenchyma and cortex and simultaneous release into the atmosphere via stomata (Davamani et al., 2020), as shown in Figure 2A. Co-existence of CH_4_-producing and CH_4_-oxidizing microbes in rice soils and control of the dynamic interplay between microbes and the environment by rice plants could provide opportunities to develop plant traits that lower net CH_4_ emissions from rice fields.

## Mechanisms of N_2O_ emissions from rice fields

The microbial conversion of nitrogen (N) results in the production of N_2_O in soil. Nitrification and denitrification are two microbial N reactions mediated by nitrifiers (e.g., *Nitrosomonas* and *Nitrobacter* spp.) and denitrifiers (e.g., facultative anaerobic bacteria like *Pseudomonas*, *Paracoccus*, and *Bacillus*) (Kuypers et al., 2018). These microbes are responsible for NH_3_-to-N_2_ transformations, with N_2_O being a by-product of these reactions and the primary cause of net N_2_O emissions, as shown in Figure 2B. Nitrifiers oxidize NH_3_ to NO_2_^−^ and then to NO_3_^−^, indirectly contributing to N_2_O production, especially under soil conditions where oxygen is limited, causing partial conversion of N to N_2_O instead of NO_3_^−^. On the other hand, during the denitrification process, the reduction of NO_3_^−^ to N_2_ and the nitrification of NH_4_^+^ under aerobic conditions result in the loss of N as N_2_O. In other words, denitrification is the progressive reduction of N oxides to gaseous products like N_2O or N_2__ in the presence of restricted O_2_, as seen in Figure 2B. This process, which is irreversible once NO is generated, results from bacteria using N oxide as a terminal electron acceptor rather than molecular O_2_ (McLain and Martens, 2005). Therefore, in an environment with low O_2_, a source of organic C is necessary for bacterial metabolism, and there must be enough NO_3_^−^ available to act as an electron acceptor.

For denitrification to occur, all three conditions must be satisfied: a C source, low O_2_, and sufficient NO_3_ (McLain and Martens, 2006). It has been observed that greater denitrification occurs at the soil surface than in deeper subsoils, owing to the higher organic input at the soil surface caused by microbial activity. Denitrifying bacteria belong to a variety of genera. Approximately 23 genera of bacteria are capable of denitrification, including *Azospirillum* (Jang et al., 2019), *Bacillus* (Yang et al., 2020), *Halobacterium* (Tomlinson et al., 1986), *Paracoccus* (Chakravarthy et al., 2011), and *Rhodopseudomonas* (Kundu and Nicholas, 1985).

Nitrification involves bacterial oxidation of NH_4_^+^ or NH_3_ through NO_2_^−^ to NO_3_^−^(Norton, 2015). Two kinds of autotrophic bacteria carry out this function. NH_3_ oxidizers accelerate the first step, which is the conversion of NH_3_ to NO_2_^−^*. Nitrosomonas* is the primary genus associated with this step, with other genera, like *Nitrosococcus*, *Nitrosospira*, and the subgenera *Nitrosolobus* and *Nitrosovibrio*, also capable of autotrophic NH_3_ oxidation. The second step is the conversion of NO_2_^−^ to NO_3_^−^, which is mediated by the genus *Nitrobacter*. Other genera are also associated with this step, including *Nitrospina*, *Nitrococcus*, and *Nitrospira* (Watson et al., 1981).

The key factors that control N_2_O emissions from rice soils are N fertilizer application rates and water management practices (Ali et al., 2021). In addition, several field studies reported that N_2_O emissions varied among rice cultivars, and the differences were unaffected by genetic variations but were instead largely influenced by N input (Wang et al., 2021). A distinct soil layer is formed in rice fields after flooding, and throughout the rice-growing season, oxidizing and reducing zones form in the cultivated layer. When N fertilizer is added to rice fields, ammonium N is nitrified, and NO_3_^−^ is formed at the water–soil interface in the oxidized layer. The NO_3_^−^ generated in the oxidized layer travels to the reduced layer and is denitrified, creating N_2_O as an intermediate product (Xing et al., 2009). The denitrification process also occurs in the soil’s subsurface saturated and above-flooded cultivated layer (Xing et al., 2002). N_2_O is produced in rice soils after intermittent flooding during the transition from wet to dry soil conditions. Moreover, winter upland crops and the rice cycle could increase with water evaporation and add to atmospheric N_2_O. Rice plants serve as a route for dissolved soil gases to move from the root zone to the atmosphere, a process that results in considerable N_2_O emissions under flooding conditions (Yan et al., 2000). Because N_2_O is a water-soluble molecule, plant roots can absorb and transmit it through leaves via the transpiration pathway. Diffusion is the primary means for N_2_O to move to the soil surface, as shown in Figure 2B. Unlike those involved in CH_4_ emissions, microbial processes involved in N_2_O production are typically related to the amount of N available in the soil, highlighting N fertilizer rate as the major driving force for N_2_O emissions.

## Impact of global warming on GHG emissions from rice fields

The average global surface air temperature is expected to increase by 1.4°C–4.4°C and atmospheric CO_2_ concentrations to reach close to 1000 ppm by the end of the 21st century (Izrael et al., 2007). Increases in atmospheric CO_2_ concentrations, global mean air temperature, and other factors related to climate change will significantly affect GHG emissions from rice fields. Rice paddies are one of the main anthropogenic sources of CH_4_, a powerful GHG, and their emissions are predicted to be affected by global warming (Qian et al., 2022). CH_4_ emissions from rice paddies are significantly influenced by agricultural practices (Qian et al., 2020). A report has shown that a 1°C increase in air temperature caused China’s rice fields to release 12.6% more CH_4_ (Qian et al., 2023). This increase probably resulted from improved C substrate availability for methanogens as well as the methanogenic activity ratio of CH_4_ to CO_2_ (Wang et al., 2018a). Furthermore, lowering the *E_h_* of the soil induces the formation of CH_4_ by decreasing the solubility of O_2_ in water or soil solution, which speeds up the rate at which microorganisms consume O_2_ and other electron acceptors. In addition, air warming could increase N_2_O emissions from rice fields by 26% (Gao et al., 2022). The increased availability of inorganic N for N_2_O generation as influenced by the acceleration of soil organic matter decomposition is probably the cause of these higher N_2_O emissions (Bai et al., 2013; Liu et al., 2020). Furthermore, heat could alter the abundance of N_2_O reductase, ammonia-oxidizing, and nitrite reductase genes in bacteria and Archaea, which might increase N_2_O emissions through effects on the soil microbial population (Wang et al., 2022). Important soil parameters that influence the output and emissions of N_2_O and CH_4_ from rice fields are mentioned in Supplemental Table 1.

One of the critical components of global warming is the rising CO_2_ concentration in the atmosphere, which has increased to a new high of 415 μmol mol^−1^, about 149% of pre-industrial (before the year 1750) CO_2_ levels (Legg, 2021). This elevated CO_2_ has a direct feedback effect on CH_4_ and N_2_O emissions by regulating the production, oxidation, and transport of these non-CO_2_ gases in rice fields (Inubushi et al., 2003; Bhattacharyya et al., 2013; Wang et al., 2018b). For example, elevated CO_2_ increased the number and activity of methanogenic bacteria, as well as the number of tillers and aerenchyma cells, leading to enhanced gas transport and high C availability from higher root biomass (Ziska, 1998; Cheng et al., 2006; Okubo et al., 2015). Whereas elevated CO_2_ promotes grain yield through higher photosynthesis and root growth (Lou et al., 2008; Lv et al., 2020), variable results have been reported regarding CH_4_ and N_2_O emissions from paddy fields. Liu et al. (2019) found that CH_4_ and N_2_O emissions from global rice fields increased by 34% and 10%, respectively (Liu et al., 2019). However, other studies indicated that greater tiller counts or larger plant biomass resulted in faster O_2_ transport into the soil, enhancing CH_4_ oxidation (Ma et al., 2010; Jiang et al., 2019c). Because of higher root growth, soil denitrification potential was improved, resulting in higher N_2_O emissions under conditions of high organic C availability (Das et al., 2013). By contrast, Sun et al. (2018) reported a reduction in N_2_O emissions under elevated CO_2_ and attributed this decline to a decrease in soil mineral N caused mainly by high plant uptake (Sun et al., 2018). In general, most studies of elevated CO_2_-induced GHG emissions in rice have been performed under short-term exposure (<5 years), and their results do not represent future CO_2_ conditions (long-term response, >10 years). The meta-analysis performed by Yu et al. (2022) demonstrated that long-term elevated CO_2_ conditions significantly decreased CH_4_ and N_2_O emissions by 18% and 43%, respectively, and that emission dynamics were associated with declines in yield and biomass over time (Yu et al., 2022). A smaller increase in total plant biomass would lead to minimal C substrate accumulation.

**Figure 3:**
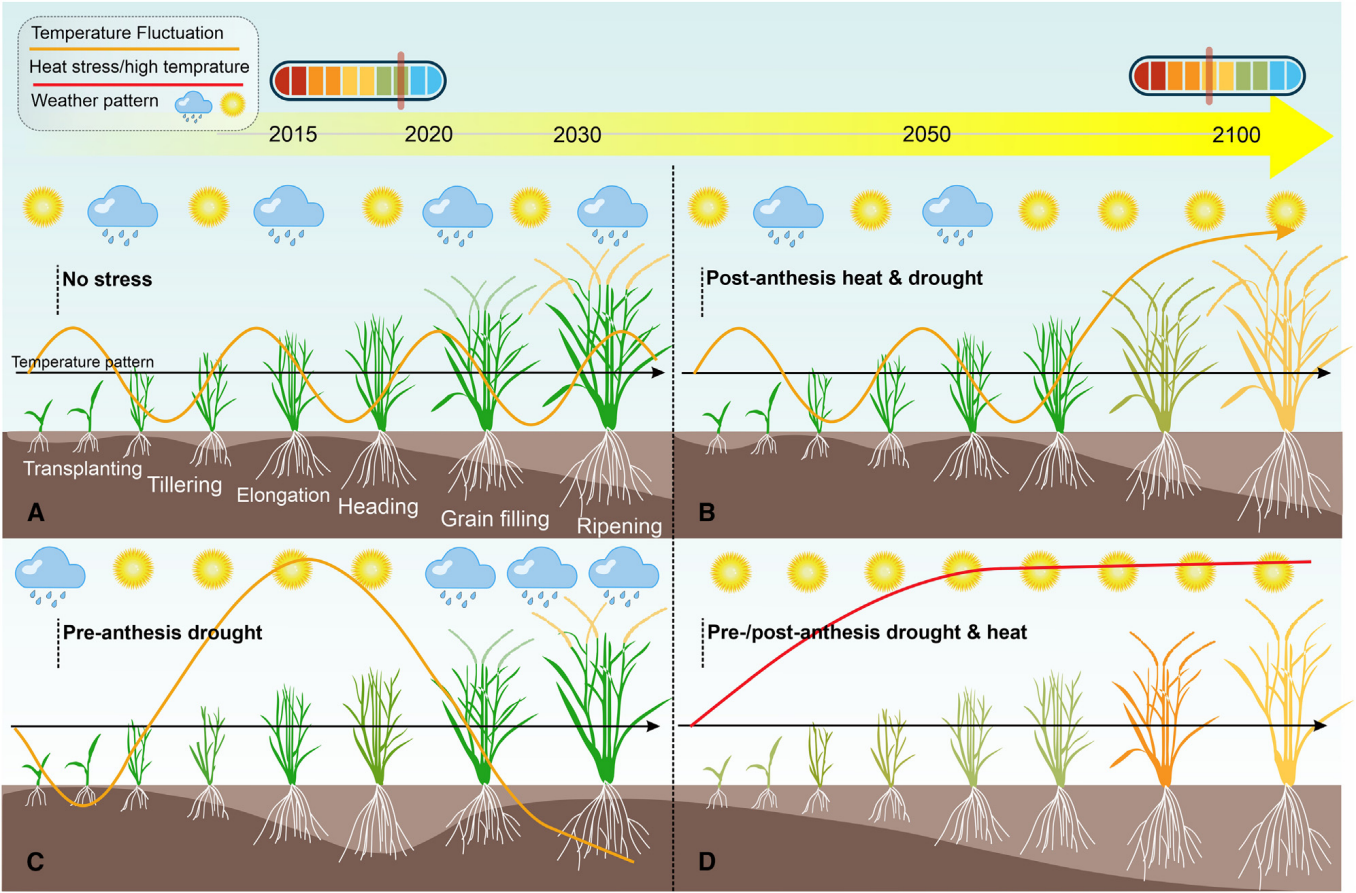
Air temperature patterns during various normal, heat, and water-stress scenarios. **(A)** No stress; yield benefit from optimized yield components and harvest index in rice. **(B)** Post-anthesis heat and drought; possible yield losses from early maturation or possible yield benefit from stay-green characteristics. **(C)** Pre-anthesis drought; severe impact on plant growth and development, leading to reduced yield. Deeper roots and/or early anthesis could help to reduce the impact of this stress. **(D)** Pre-/post-anthesis drought and heat; significant impact on plant morphology, physiology, and development, causing dramatic yield reduction.

Given the reduction in grain yields under the scenario of rising CO_2_, rice yield is also significantly affected by higher air temperatures. According to research performed by the International Rice Research Institute (IRRI) in the Philippines, an increase of 1°C in nighttime air temperature results in a 10% decrease in rice grain yield. An increase in atmospheric CO_2_ and a 1°C rise in temperature were shown to increase yield-scaled GHG emissions by 31.4% and decrease rice output by 11.8% (Van Groenigen et al., 2013). In previous studies, the enhancement of GHG emissions was attributed to additive effects of elevated CO_2_ and air temperature (Ziska, 1998; Tokida et al., 2010). Importantly, when air temperature rises by 1°C above historical levels, the global mean crop yields of the main staple foods (including rice) are expected to drop by 3%–10% (Wang et al., 2018a), as shown by different climate patterns and elevated temperatures in Figure 3. Although global climate changes have variable effects on GHG emissions, these changes increasingly challenge modern rice production.

## Mechanisms and processes driving the effects of mitigation practices on GHG emissions

Three primary crop-management parameters (i.e., irrigation water, soil organic matter, and fertilizer) and the use of low-GHG-emitting rice varieties can effectively decrease CH_4_ and N_2_O in rice fields. These interventions directly affect soil microbial activity by changing the availability and dynamics of microbial growth substrates, namely, carbon and N. Critical strategies for decreasing GHG emissions from rice fields are described below.

### Irrigation water management

**Figure 4:**
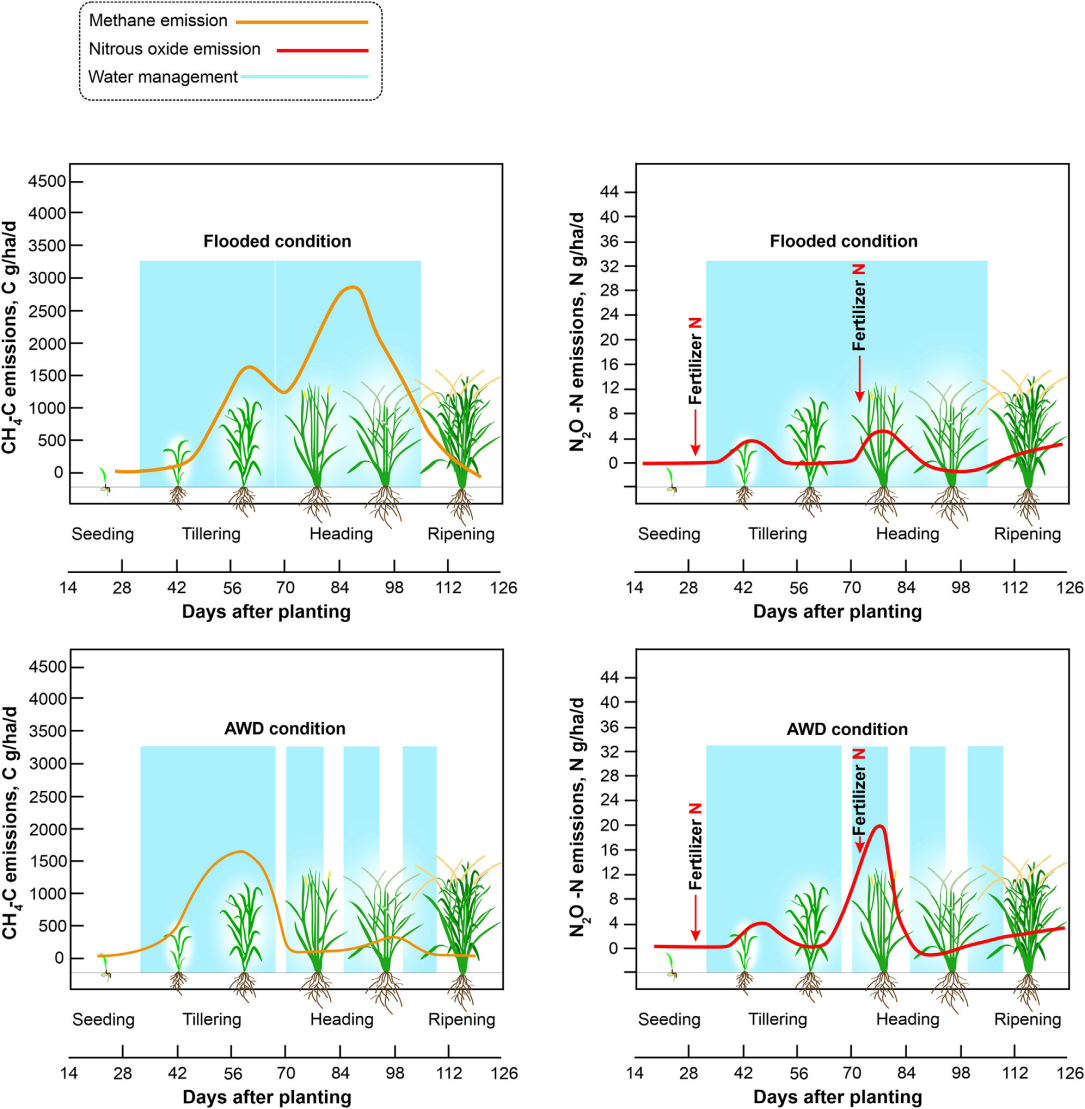
Dynamics of GHG emissions from drill-seeded rice fields. The trade-off between CH_4_ and N_2_O emissions in flooding and non-flooding (AWD) practices. Shaded portions indicate flooded conditions. Orange and red curves represent the patterns of CH_4_ and N_2_O, respectively, under different water-regime practices.

A primary strategy for decreasing CH_4_ emissions from paddy fields is irrigation water management. Water management changes soil moisture and soil *E_h_*. These two factors have a substantial impact on how quickly GHGs are released and consumed (Wang et al., 2017). Non-continuous flooding (NCF) techniques, such as midseason drainage, intermittent irrigation, and alternate wetting and drying (AWD) (Minamikawa et al., 2019), usually decrease the presence and activity of methanogens (Qian et al., 2023). Increases and decreases in CH_4_ emissions during rice growth depend largely on the duration of flooding and rice phenology. Figure 4 shows a typical CH_4_ emission profile for drill or dry seeding under continuously flooded irrigation. Here, CH_4_ emissions are low and close to zero during the early growth stage because soils are not saturated and aerenchyma cells are not yet fully developed. As rice plants grow and reach the vegetative stage, CH_4_ emissions increase, peak around heading, and then decline toward physiological maturity. A sharp decline in CH_4_ emissions occurs when flooding is disrupted around the reproductive stages, such as during the dry-down event in AWD irrigation. Low CH_4_ emissions may extend during this stage if fields undergo frequent dry and wet cycles.

As soil O_2_ concentrations and *E*_*h*_ increase during a dry-down event, methanotroph activity and abundance increase, stimulating CH_4_ oxidation while inhibiting methanogens. Different irrigation techniques in NCF practice, such as scheduled, midseason, intermittent, AWD, and furrow irrigation, have the potential to minimize N_2_O and CH_4_ emissions without compromising grain yield. The frequency, timing, and intensity of soil drying duration could be optimized to maximize reductions in GHG emissions. For example, the total number of days without flooding is correlated with the effect of NCF on CH_4_ emissions; on average, single and several drying episodes decrease CH_4_ emissions by 33% and 64%, respectively (Jiang et al., 2019b). The Guidelines for National Greenhouse Gas Inventories, published by the Intergovernmental Panel on Climate Change in 2006, estimated a median 48% reduction in CH_4_ emissions compared with the baseline of transplanted puddled fields. CH_4_ emissions from rice were found to be 43% lower using AWD irrigation rather than conventional, continuously flooded irrigation when combined with water conservation (Sander et al., 2016). On the other hand, N_2_O emissions from continuously flooded rice systems are often negative or low throughout the growing season (Perry et al., 2022), because nitrification and denitrification activities predominantly occur during re-flooding and drying of soil (Bouwman, 1998). N_2_O peaks generally occur after the addition of N fertilizer during the early stage of rice growth or the midseason stage, when another dose of N fertilizer is applied (Figure 4). Under NCF, soil O_2_ concentrations increase, and N-converting microorganisms become more active, increasing N_2_O emissions (Hou et al., 2000; Islam et al., 2018).

### Soil organic matter management

The second main management parameter is organic matter. Farmyard manure, green manure, and crop residue are conventional products used by farmers to manage soil fertility. The community composition of methanogens and methanotrophs changes when organic matter is added over an extended period of time. These changes are indicated by the relative abundance of hydrogenotrophic and acetoclastic methanogens (Zhang et al., 2018; Raheem et al., 2022) and by the abundance of methanotrophs that may prefer high CH_4_ concentrations (Yang et al., 2022a). Different straw management practices cause significant changes in soil organic carbon composition and dynamics (Jiang et al., 2019a; Yang et al., 2021). However, because straw incorporation increases soil organic carbon sequestration in the long term, the addition of organic matter to rice fields may have a net climatic impact (Liu et al., 2014). Crop yields have frequently been reported to increase with soil organic carbon (SOC) content (Oldfield et al., 2022). Farm strategies based on soil organic matter management can have conflicting effects on yield and emissions. However, by considering the net carbon balance of the system, techniques such as straw removal, the use of varieties with high root growth, and fields with high SOC may result in decreased emissions. Potential tradeoffs may be considered for environments with low SOC, as straw incorporation is desirable in combination with other practices. Some reports have suggested that a high-yielding variety with more extensive root growth can increase emissions. This interactions may lead to a net positive carbon gain at the system level by limiting the increase in emissions while enabling more significant gains in SOC sequestration.

### Fertilizer management

CH_4_ and N_2_O emissions can be indirectly and directly affected by fertilizer N management, leading to variations in emissions. Previous studies have reported that fertilizer N can increase, decrease, or have no effect on CH_4_ emissions (Cai et al., 2007; Shang et al., 2011; Yao et al., 2012). However, a recent meta-analysis showed that the influence of N fertilizer depends largely on input rate, with low to moderate N rates increasing CH_4_ emissions and excessive N rates decreasing CH_4_ emissions (Linquist et al., 2012). In particular, N fertilization increases the activity of methanogens and speeds up the breakdown of organic matter, which most strongly increase CH_4_ emissions in acidic soils (Tang et al., 2024).

The application of mineral N has also been reported to produce higher CH_4_ emissions than those in low input systems without N application (Schroeder et al., 2013). A decrease in the amount of CH_4_ oxidation occurred as a result of CH_4_ monooxygenase binding and reacting with NH_4_^+^ (Gulledge and Schimel, 1998). By contrast, N_2_O emissions are related to the time of N fertilization and water management practices. High N_2_O emissions have been reported in fields with intermittent or midseason dry events or with N application rates above optimal levels (Cai et al., 1997; Zou et al., 2005). Furthermore, increased crop growth in response to N fertilizer increases shoot and root development, which in turn increases substrate availability for methanogens (Schimel, 2000). Rice yield and the type and rate of N fertilizer are also related to emissions from rice fields. Subsurface N application, enhanced-efficiency N fertilizers, and optimal N rate have been reported to reduce GHG emissions. Better land use planning, effective field management techniques, less land disturbance, direct planting, and climate-smart water management practices might also minimize CH_4_ and N_2_O emissions.

### Effects of rice varieties on GHG emissions

Emissions vary significantly among high-yielding rice cultivars, likely because of differences in CH_4_ production, CH_4_ oxidation, anatomical characteristics, and gas transport capacities (Watanabe et al., 1995; Wang et al., 1997, 2000; Yang et al., 2009; Qin et al., 2015). Rice plants have two main strategies to control their CH_4_ emissions. The first of these processes involves the rhizodeposition of rice plants, which supplies 40%–60% of the organic C as CH_4_ substrate to methanogens starting at the booting stage (Watanabe et al., 1999; Yuan et al., 2012). The second mechanism is the diffusion of atmospheric O_2_ into the rhizosphere of rice plants through root aerenchyma, which stimulates CH_4_ oxidation (Conrad, 2007). Varietal effects have been demonstrated through field screening for CH_4_ emissions among different rice types and varieties. These effects were reported as non-significant with the limited sets of varieties tested. However, the varietal effects were observed consistently when integrated with management and environment.

A meta-analysis to quantify the effects of rice varieties on the GWP of GHG emissions at the yield scale in China revealed that *indica* rice varieties had a significantly higher yield-scaled GWP (1101.72 kg CO_2_ equiv Mg^−1^) than *japonica* rice varieties (711.38 kg CO_2_ equiv Mg^−1^) (Zheng et al., 2014). This difference may be attributed to varietal differences in root exudation, organic matter decomposition in flooded soils, and interactions with soil microbiota, which result in various levels of CH_4_ emissions. In addition, rice varieties can affect N_2_O emissions through differences in N use efficiency and N cycling in the soil. Also, a higher harvest index and productivity per unit day could reduce GHG emission relative to that of longer-duration varieties (Smith et al., 2013). Jiang et al. (2017) screened 33 rice cultivars and found that those with lower emissions were high-yielding cultivars with higher biomass and enhanced root porosity (Jiang et al., 2017). Aerobic rice varieties were reported to release 80%–85% less CH_4_ into the environment and to have a reduced carbon footprint, as they are grown under NCF conditions while at the same time contributing to increased carbon assimilation through greater crop growth and yield potential (Parthasarathi et al., 2012; Sandhu et al., 2013; Sritharan et al., 2015). Furthermore, studies have shown that lines that are robust to drought and show minor yield loss under different water regimes have low CH_4_ emissions (Sander et al., 2015). Growth duration also has a significant effect on seasonal emissions; varieties with a shorter growth duration have 25%–30% lower emissions overall compared with medium- and late-maturing varieties with similar daily emission rates.

Over the last decade, significant efforts have been made to breed climate-resilient varieties. This is a critical strategy for addressing the effect of climate change on rice production (Haefele et al., 2016; Atlin et al., 2017). No genetic engineering program is currently focused on breeding low-GHG-emitting varieties, although hypotheses about the features that contribute to differences in CH_4_ emissions across genotypes have been proposed. For instance, cultivars that demonstrate ozone tolerance, improved N use efficiency, and higher water use efficiency have been shown to generate less CH_4_. Among the traits of interest are a strong root-oxidizing capacity, a high harvest index, and fewer unproductive tillers (Wang et al., 1999). Varieties with lower respiratory losses will potentially have lower GHG emissions (Chauhan and Mahajan, 2016). Similarly, varieties with lower root exudates and less aerenchyma, may also have reduced emissions (Weller et al., 2018). As a result, breeding programs that leverage these findings, e.g., by creating genotypes with a larger rhizosphere and less carbon release from the root zone, can contribute to transforming rice systems for lower carbon emissions. It is worth noting, however, that advances in the creation of cultivars tolerant to water-scarce environments have enabled the development of high-yielding varieties adapted for Direct seeded rice. These varieties facilitate the scaling of rice systems under aerobic conditions and NCF water management, leading to lower CH_4_ emissions. In addition, efforts to develop highly productive genotypes with shorter growth durations continue in current breeding programs.

## Mitigation through wide-scale adoption of improved low-GHG-emitting rice hybrids

**Figure 5:**
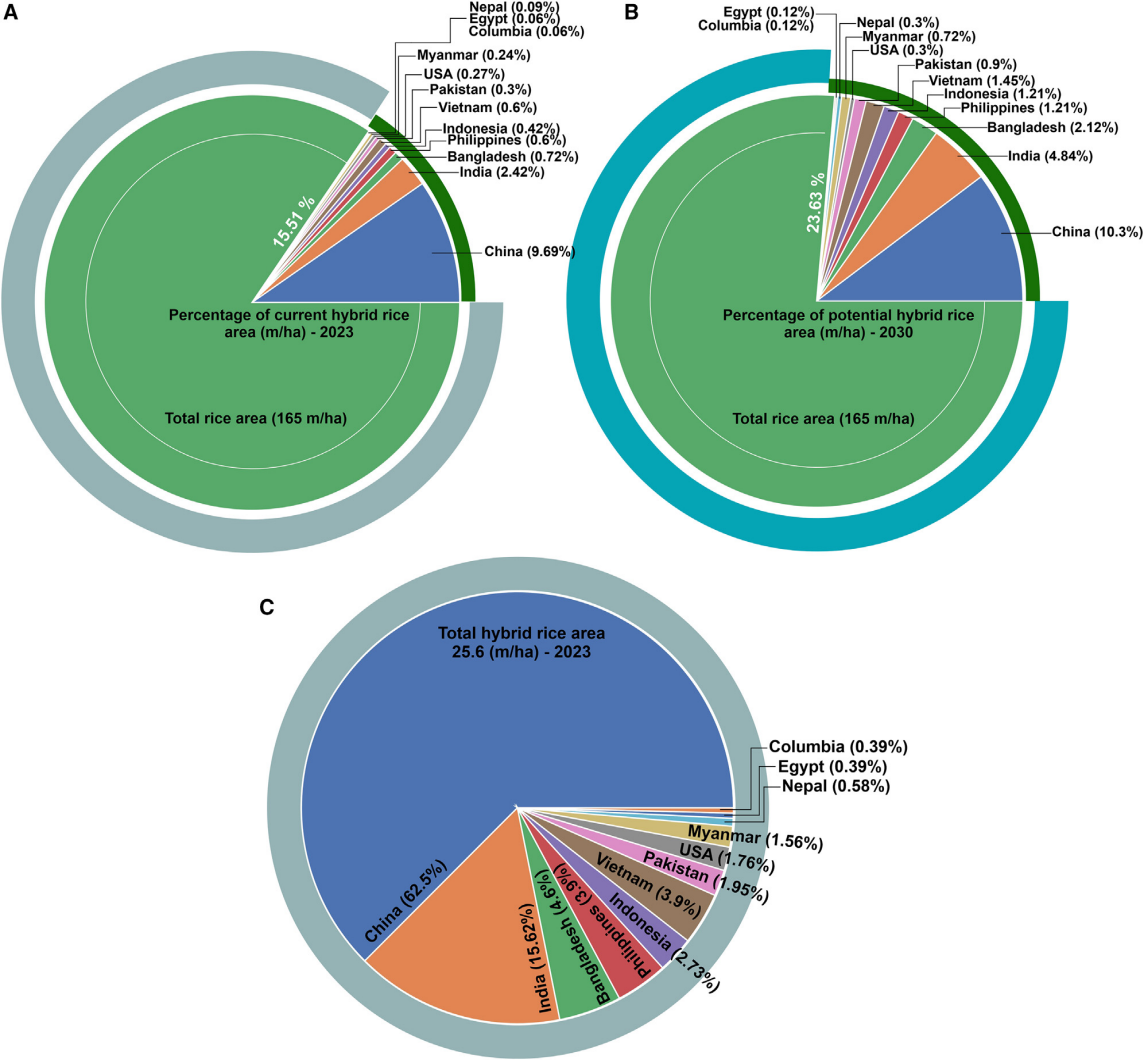
Current and predicted areas under hybrid rice cultivation. **(A)** Percentage of current hybrid rice area in m/ha (2023). **(B)** Potential hybrid rice area in m/ha (2030). **(C)** Percentages of the current hybrid rice area accounted for by top hybrid-rice-growing countries.

According to the latest report in 2022, approximately 165 million hectares (m/ha) of land are under rice cultivation across the world, and 15.51 m/ha of these were under hybrid rice cultivation in 2024. According to the IRRI’s hybrid rice program, the estimated area under hybrid rice cultivation by 2030 will be approximately 23.63 m/ha, as shown in Figure 5A and 5B, respectively. Figure 5C also presents the percentage of hybrid rice cultivation area that is accounted for by each of the top hybrid-rice-growing countries.

The amount of CH_4_ emitted per hectare is approximately 300 kg CH_4_ ha^−1^ season^−1^ (Wassmann et al., 2000a), equivalent to approximately 49.5 Mt CH_4_ per season worldwide. Studies have shown that paddies with high-yielding hybrid rice varieties produce approximately 19% lower CO_2_ emissions than those with inbred varieties under the same growth conditions (Taghavi et al., 2017). By extrapolating this value to the total area under hybrid rice cultivation in 2024, the total CO_2_ equiv emissions will be approximately 102.5 Mt per season for the total area under hybrid rice cultivation.

Hybrid rice technology is also a viable approach for obtaining grain yields 30% higher than those of the best inbred varieties (Wang et al., 2024). Many of the high-yielding hybrid rice varieties have a shorter growth duration than inbred varieties. Rice hybrids have higher productivity per day than inbred varieties and need less time in the field with reduced irrigation levels. This makes hybrid rice highly effective in reducing GHG emissions from rice fields. Interestingly, several climate-resilient, shorter-duration rice hybrids have recently been developed at the IRRI, with a duration of <110 days and a grain yield exceeding 10 t/ha. This directly effects the significant reduction in GHG emission compared with that of inbred varieties. It is important to know that certain hybrids produce lower CH_4_ emissions, and researchers need to identify them. Smartt et al. (2018) clearly demonstrated that CH_4_ emissions were significantly lower from three hybrid rice varieties (CLXL729, XL753, and CLXL745) than from the inbred variety RoyJoy. These hybrids have the potential to mitigate CH_4_ emissions from rice production on silt loam soils in the mid-southern United States. In another study, a hybrid rice (CLXP4534) produced significantly lower CH_4_ emissions than inbred varieties under continuous flooding conditions (Simmonds et al., 2015). From Table 1, it is very clear that there are genotypic differences among the hybrids. Certain hybrids, like RT7521 FP, CLXL745, and CLXP4534, were found to emit less CH_4_ than other hybrids and inbreds. Therefore, it is essential to breed and identify parental lines that emit less CH_4_ in order to develop low-CH_4_-emitting hybrids.

**Table 1:** Relative performance and GHG emissions of rice inbreds and hybrids under different irrigation practices in the United States and Asian countries

Irrigation practice	Country	Cultivar	Type	Methane (CH_4_)	Nitrous oxide (N_2_O)	Global warming potential (GWP)^a^		Grain yield	Reference
				kg CH_4_-C ha^−1^ season^−1^	kg N_2_O-N ha^−1^ season^−1^	kg CO_2_ equiv ha^−1^ season^−1^	kg CO_2_ equiv t^−1^ season^−1^	t ha^−1^	
				–	–	Area-scaled	Yield-scaled	–	
Continuously flooded	United States	Francis^b^	inbred	60	0.10	2102	332	6.2	Simmonds et al., 2015
		Jupiter^b^	inbred	72	0.04	2397	345	6.9	
		Sabine^b^	inbred	65	0.11	2286	391	5.8	
		**CLXP4534**	**hybrid**	**25**	0.17	981	140	7.0	
		CLXL745	**hybrid**	**56**	0.02	1899	232	8.2	
Continuously flooded	United States	RoyJoy^b^	inbred	75	nd	nd	nd	9.8	Smartt et al., 2018
		CLXL729	**hybrid**	**55**	nd	nd	nd	9.8	
		CLXL745	**hybrid**	**49**	nd	nd	nd	9.8	
		XL753	**hybrid**	**53**	nd	nd	nd	12.6	
Continuously flooded	United States	CL151^b^	inbred	120	0.17	4562	536	8.0	2019 technical reports to RiceTec and USDA
		XP753	**hybrid**	115	0.10	4333	436	10	
		XP760	hybrid	129	0.08	4848	555	10	
		CLXL745	**hybrid**	106	0.08	4003	439	9.0	
Continuously flooded	United States	CLL15^b^	inbred	74	0.70	2984	352	10	2022 technical reports to RiceTec and USDA
		RT7321	**hybrid**	82	0.65	3239	265	12	
		**RT7521 FP**	**hybrid**	56	0.71	2332	198	12	
Alternate wetting and drying (AWD)	United States	CL151^b^	inbred	39	0.50	1682	191	8.0	2019 technical reports to RiceTec and USDA
		XP753	**hybrid**	**37**	0.15	1434	156	10	
		XP760	**hybrid**	**38**	0.10	1454	156	11	
		**CLXL745**	**hybrid**	**21**	0.17	872	101	9.0	
Furrow irrigation	United States	CLL15^b^	inbred	14	1.11	979	117	8.5	2022 technical reports to RiceTec and USDA
		RT7321	**hybrid**	**12**	0.88	791	69	12	
		**RT7521 FP**	**hybrid**	**5.7**	0.72	513	44	12	
Continuously flooded	India	Monohar Sali^b^	inbred	140	nd	nd	nd	3.5–6	Gogoi et al., 2008
		Betguti Sali^b^	inbred	119	nd	nd	nd	3–3.5	
		Peoli^b^	inbred	107	nd	nd	nd	3.5–4	
		IR-36^b^	inbred	66	nd	nd	nd	3–5	
Continuously flooded	China	Zhongzhuo^b^	inbred	17	nd	nd	nd	7.7	Wang et al., 2000
		Jingyou^b^	inbred	37	nd	nd	nd	6.8	
		Zhongzhua^b^	inbred	33	nd	nd	nd	6.9	
		IR72^b^	inbred	24	nd	nd	nd	4.5	
Continuously flooded	China	Zhongfu 906^b^	inbred	71	nd	nd	nd	5.2	Lu et al., 2000
		Xiusui 11^b^	inbred	75	nd	nd	nd	5.1	
		Chungjiang 06^b^	inbred	137	nd	nd	nd	5.2	
		II-you 1568	**hybrid**	**84**	nd	nd	nd	4.9	
		**Jin23a/71**	**hybrid**	**67**	nd	nd	nd	4.9	
		Shanyou 10	**hybrid**	**125**	nd	nd	nd	5.5	
Continuously flooded	Philippines	IR65597^b^	inbred	4.5	nd	nd	nd	1.5	Wassmann et al., 2000b
		PSBRc14^b^	inbred	4.5	nd	nd	nd	3.1	
		**Magat**	**hybrid**	**3**	nd	nd	nd	5.1	
		IR72	inbred	6	nd	nd	nd	3.1	
Continuously flooded	China	Huanghuazhan^b^	inbred	240	nd	nd	nd	25^c^	Liao et al., 2019
		**Rongyouhuazhan**	**hybrid**	**188**	nd	nd	nd	27^c^	
Continuously flooded	Indonesia	Ciherang^b^	inbred	278	nd	nd	nd	5.5	Kartikawati et al., 2019
		Sembada 989	hybrid	239	nd	nd	nd	4.8	
		Sembada 168	hybrid	304	nd	nd	nd	5.3	
		Mapan 05	hybrid	442	nd	nd	nd	6.0	
		Arize Gold	hybrid	495	nd	nd	nd	6.2	
		Intani	hybrid	398	nd	nd	nd	5.7	
		Hipa 8	hybrid	399	nd	nd	nd	5.2	
		Hipa 18	hybrid	335	nd	nd	nd	5.2	
		Hipa 19	hybrid	343	nd	nd	nd	5.0	

nd, no data.

a GWP (area-scaled) was computed using IPCC 2021 conversion factors of 273 and 28 over a 100-year time horizon for N_2_O and CH_4_, respectively, whereas GWP (yield-scaled) was calculated as the ratio of GWP to grain yield.

b Inbred cultivars. ^§^Pot experiment in which grain yields were extrapolated from g plant^−1^.

Hybrid varieties have greater root porosity than inbred cultivars and can therefore transport more O_2_ into the soil for methanotrophs, promoting greater CH_4_ oxidation (Ma et al., 2010; Kim et al., 2018). Previous studies have demonstrated that CH_4_ emissions vary significantly among high-yielding rice varieties, likely owing to differences in CH_4_ production, CH_4_ oxidation, anatomical characteristics, and gas transport capacities (Watanabe et al., 1995; Wang et al., 1997, 2000; Yang et al., 2009; Qin et al., 2015). With more evidence supporting reduced CH_4_ emissions from high-yielding rice varieties, there is increasing interest in the use of hybrid varieties to develop strategies for reducing CH_4_ emissions while simultaneously producing higher grain yields. In addition, because rice hybrids have higher root biomass and a deeper root system, they help with sequestration of carbon deeper in the soil. It is also possible that the robust root systems of hybrids closer to the soil surface may be able to attract methanotroph bacteria to break down CH_4_ into CO_2_ and water (Figure 3). However, the molecular and physiological mechanisms that underlie reduced GHG emission by hybrids under flooded and non-flooded conditions have yet to be studied intensively.

Several studies have shown that certain low-GHG-emitting hybrid varieties grown under continuous flooding and/or NCF consistently had lower CH_4_ emissions than inbred cultivars (Ma et al., 2010; Smartt et al., 2016; Brye et al., 2017; Liao et al., 2019). Field studies performed across the United States and Asia demonstrated that there was, on average, a 25% decrease in total CH_4_ emissions in some high-yielding hybrids compared with those in inbred varieties under continuous flooding irrigation (Table 1). CH_4_ emissions, especially in low-GHG-emitting, high-yielding hybrid varieties, declined by 52% relative to inbred cultivars under AWD/furrow irrigation (Table 1). Also, according to these United States studies (Table 1), there was no difference in average total N_2_O emissions between inbred and hybrid varieties and irrigation practices at recommended fertilizer N rates, which averaged 0.35 kg N_2_O-N ha^−1^ season^−1^. Expressing the GWP of CH_4_ and N_2_O emissions relative to grain yield, as opposed to total area, provides a better assessment of the agronomic and environmental benefits of GHG mitigation strategies. The results in Table 1 show that the yield-scaled GWP of some low-GHG-emitting hybrids decreased by 34% and 55% for continuous flooding and NCF irrigation, respectively. Here, the optimal cultivar response when considering a win–win strategy to mitigate GHG emissions through varietal selection is to have the lowest yield-scaled GWP with the highest grain yield and the lowest area-scaled GWP.

### Carbon sequestration capacity of hybrid rice

On a global scale, rice soils (0–100 cm) contain an average of 108 Mg SOC ha^−1^, corresponding to 1.2% of the worldwide SOC pool (Smith et al., 2007; Liu et al., 2021a, 2021b). Although rice soils cover less than 9% of the total global cropland area, these soils retain more than 14% of their SOC stocks (Woodwell, 1984; FAO, 2017; Liu et al., 2021b), suggesting that rice soils have more SOC than upland agricultural soils (Pan et al., 2004; Wu, 2011). Unlike cropland soils, rice soils have high SOC because they are under anaerobic conditions due to periodic flooding and long-term puddling (Qiu et al., 2018). Anaerobic conditions slow the rate of organic matter decomposition, which, in turn, increases soil C accumulation compared with upland soils (Wang et al., 2015; Wei et al., 2021). Agronomic practices that increase biomass production, increase crop residue input, and slow the production of respiratory CO_2_ can increase SOC reserves, thereby sequestering carbon (Lal, 2004b; Wang et al., 2015). However, although rice fields store more SOC than the global average, an increase in soil C stocks does not always lead to C sequestration if there is no net removal of CO_2_ from the atmosphere. There are limits to the amount of C that soils can sequester, and this soil C saturation is driven by soil texture, aggregation, and, to some extent, the biophysical composition of the organic input (Six et al., 2002). According to Lal (2004a), the attainable soil C capacity is only 50%–66% of the potential soil capacity. Soils with a large C saturation deficit sequester more C than soils close to saturation.

Many rice fields, particularly in Asia, are under long-term puddled rice cultivation and have various amounts of soil C stocks, leading to various degrees of C sequestration potential (Ma et al., 2010, 2021; Kalbitz et al., 2013). Changes in C pool size could strongly affect atmospheric CO_2_ concentrations. The amount of soil organic C in rice paddies is directly related to the decomposition of soil organic matter, which mainly produces CO_2_. CO_2_ emissions from agriculture contribute <1% to the total global C budget because CO_2_ emissions are largely offset by high rates of net primary productivity and CO_2_ uptake by crops (Friedlingstein et al., 2020). Researchers have agreed that changes in SOC over time reflect the net balance between soil respiration and C fixation in cropland (Stewart et al., 2007; Deng et al., 2024). Hybrid rice, through its higher yield performance, may accelerate the fixation of CO_2_ through a high photosynthetic rate and biomass production, thereby facilitating and improving the C sequestration capacity of rice paddies. Although hybrid rice affects C sequestration, no studies have demonstrated this effect for SOC.

### Future hybrid rice breeding strategies for low carbon emissions

Despite the enormous amount of research on linking various high-yielding rice cultivars to lower CH_4_ emissions (Table 1), the net effects of hybrid varieties on CH_4_ and N_2_O emissions and the underlying mechanisms involved in decreasing GHG emissions are largely unknown. Many studies have speculated that hybrid rice varieties have lower CH_4_ emissions because of different genotypic traits associated with CH_4_ oxidation, such as photosynthetic C partitioning, a more robust root system, greater gas transport capacity, efficient metabolic activity, and plant architecture (Table 1). Recently, several studies have tried to explain the fundamental processes that control CH_4_ emissions under emerging irrigation technologies such as intermittent flooding. These studies have focused on complex interactions between high-yielding hybrid varieties and microbes under drained conditions to better understand the feedback of the aerobic cycle on CH_4_ emissions and rice productivity (Edwards et al., 2015; Fernández-Baca et al., 2021). For example, Santos-Medellín et al. (2021) reported a compositional shift characterized by an increase in Actinobacteria (e.g., *Streptomyces*) in the endospheric communities of the rice root microbiota, which affected root microbial recovery during a prolonged dry cycle. Liechty et al. (2020) reported that the root microbiome of the high-CH_4_-emitting inbred rice variety Sabine, compared with that of the low-CH_4_-emitting hybrid rice variety CLXL745, was characterized by both methanogens and other bacterial groups associated with fermentation, iron, and sulfate reduction and acetogenesis, processes that support methanogenesis. This alteration of microbial communities driven by the aerobic cycle requires further research because microbial communities in rice fields exhibit considerable variation, and our current understanding of CH_4_ cycling is based on cultured strains and known groups of soil microbiota that may not be viable across diverse rice environments (Conrad, 2007; Zhang et al., 2017). Under current and predicted climatic conditions, the development of climate-resilient hybrid cultivars relies on acclimating these new plant types to changes in management practices and growth environments. One primary trait needed to improve hybrid response to climate-driven abiotic stresses and, at the same time, reduce GHG emissions is the efficient use of water and N fertilizer. As discussed previously, the implementation of dry-down conditions during rice growth has been recognized to reduce >50% of total CH_4_ emissions. However, the field performance of major rice hybrid germplasms under non-flooding irrigation practices showed that the recurring water stress commonly observed in intermittent flooding may reduce grain yield by 7%–89%, depending on the severity of the dry cycle and the duration of drought stress (Villa et al., 2012; Monkham et al., 2015; Torres and Henry, 2018; Johnson et al., 2023). Although biochemical, plant architectural, and physiological traits have been linked to grain production under water stress, there are still no consistent correlations between component traits (root biomass, stomatal conductance) and grain yields (Dixit et al., 2014; Vikram et al., 2015). This observation is not uncommon, because the response of rice plants to abiotic stress is complex and involves hundreds of genes. Recent studies have suggested that rice crops may sustain high grain yield production under water stress if certain biochemical properties (accumulation of more carbohydrates in leaves, maintenance of stomatal conductance and photosynthetic rates, and better regulation of canopy temperature) are achieved (Fukuda et al., 2018; Barnaby et al., 2019; McClung et al., 2019). Here, it appears that a detailed understanding of the response of rice plants to restrictive drought conditions, such as reoccurring dry events and the extent of dryness, is a prerequisite for developing hybrids tolerant to water stress. Breeding of new rice hybrids should also consider the interaction between plant productivity and efficient use of N in soil. N is a critical constituent of plant cells and chlorophyll and promotes the rapid growth of panicles and grains. Because N is the primary nutrient for growth and yield performance, slight N deficiencies can reduce rice growth and productivity. When fields undergo a dry cycle, soil N can be lost through denitrification, immobilization, and fixation, becoming unavailable for plant uptake (Buresh et al., 2008). In addition, drying a flooded rice field can increase N_2_O emissions through denitrification and nitrification, creating a trade-off between CH_4_ and N_2_O emissions. Field studies have reported a significant increase in N_2_O emissions with excessive N fertilizer application and inefficient irrigation water management (Adviento-Borbe et al., 2013; Lahue et al., 2016). Therefore, efforts to identify new genes responsible for the low-CH_4_-emission trait should include genetic traits that can produce more grains under suboptimal soil N content. This could involve the expression of genes responsible for the high-affinity transport system, which functions under low N concentrations (<250 μM) and uses Nitrate Transporter 2 (NRT2) and Ammonium Transporter 1 (AMT1) for the uptake of NO_3_^−^ and NH_4_^+^, respectively (Li and Zhang, 2009; Dechorgnat et al., 2019). Another strategy that might enable hybrid rice to resist the effects of climate change is to introduce new genes that reduce net CH_4_ emission by enhancing CH_4_ oxidation through low amounts of root C exudate production. Scientists have long acknowledged that the primary factor controlling CH_4_ emissions in lowland rice is the presence of the plant itself. Recent studies reported that the contribution of root organic C to CH_4_ production was 41% at tillering and about 60% from booting to the maturity stage, demonstrating that rice roots produce organic acids and carbohydrates that are significant substrates for the formation of CH_4_ in rice paddies (Lu and Conrad, 2005; Yuan et al., 2012; Leichty et al., 2021). Several studies have reported that acetate, ethanoic acid, malic acid, citric acid, and succinic acid from root exudation or substrate fermentation are the main precursors for CH_4_ production (Wassmann et al., 1998; Chidthaisong et al., 1999; Moscôso et al., 2019; Qi et al., 2024). However, the primary source for methanogenesis among all major root-released C substrates is still unidentified. One primary focus for the development of new plant types is a better understanding of the underlying mechanisms and the target C substrates that directly control CH_4_ oxidation and production in rice soils and how these substrates are altered under a changing climate. As described above, the successful inclusion of hybrid rice varietal selections in multiple mitigation management practices requires identification of the genes responsible for the low-CH_4_-emitting, high-yielding trait and the expression of this trait in new and improved hybrid genotypes. An essential consideration for breeding of a climate-resilient hybrid is the viability of the new plant type under agricultural field conditions and its acclimation to stressful growing conditions associated with climate change (i.e., salinity, drought, high CO_2_ content, and high nighttime air temperature).

## Smart plant breeding programs for GHG mitigation

Rice breeding programs must identify rice germplasms with lower GHG emissions, and such materials must be used for the entire rice breeding pipeline, especially targeting transplant conditions. Pre-breeding efforts must identify potential donors and place target genes relevant to different market segments into elite parental backgrounds. Varieties must be bred for short duration with lower CH_4 emissions_ under both transplanted and direct-seeded conditions. Target traits should include higher general combination ability, higher outcrossing features, earliness, anaerobic germination tolerance, seedling vigor, nutrient use efficiency, water use efficiency, abiotic stress tolerance to drought and heat, and resistance to nematodes, blast, bacterial blight, and brown plant hopper. Breeding within the heterotic pools should follow a genomic selection approach, keeping the crosses limited to target traits related to low GHG emissions. Parental lines with lower GHG emissions will be helpful for developing a series of high-yielding, short-duration, low-carbon-footprint rice hybrids. Rice breeding must also go hand in hand with crop management practices that influence the production of GHGs in the rice environment. Identification of low-GHG-emitting hybrids is essential to maximize the gains for a given set of management practices. Furthermore, modification of the management system can also provide robust opportunities for mitigation options. The correct choice of low-GHG-emitting hybrids that are high yielding and have a short duration is of primary importance. It is vital to augment this choice with proper agricultural and management practices, such as the timing of irrigation schedules, management of organic additives, appropriate amounts and rates of N fertilizers, tillage procedures, cropping regimes, etc., to mitigate N_2_O and CH_4_ emissions from rice fields. The emergence of new technologies such as AI, genome editing, and genomic selection could bring about a revamped workflow for obtaining new types of rice varieties with minimal changes to cultural practices and could promote cost-effective farm operations while limiting the production and emission of N_2_O and CH_4_ from rice fields and maintaining high yield performance.

### Genome editing strategies to target genes that control low GHG emissions

Where large-scale phenotyping facilities exist, the identification of candidate genes and quantitative trait loci could enable the investigation of plant features that contribute to mitigating CH_4_ emissions. Association mapping is another method for identifying genomic regions underlying a specific region linked to low CH_4_ emissions or associated qualities in available germplasm resources. The genetic and allelic variations between rice lines with high and low CH_4_ emissions can be examined, despite the lack of genomic data on regions associated with low CH_4_ emissions.

Researchers are now looking into new methods for precise and rapid genomic modifications to increase crop production and protect crops from various challenges (Taranto et al., 2018). The most effective method for modifying the plant genome with sequence-specific nucleases is genome editing (GE). The tremendous capacity of GE for crop development to combat food insecurity and create a worldwide climate-smart agricultural system is unmatched (Liu et al., 2013). GE technologies have had a significant impact on plant breeding techniques. The site-specific endonucleases used in GE technologies include CRISPR-Cas9, transcription activator-like effector nucleases, and zinc-finger nucleases (Zhu et al., 2017). The CRISPR-Cas9 system is emerging as the most effective GE technique because it is affordable, quick, and accurate. It enables site-specific editing inside the genome, in contrast to GE tools based on zinc-finger nucleases and transcription activator-like effector nucleases (Raza et al., 2019). GE could be used as a rapid breeding approach for editing critical genes that reduce GHG emissions in rice and the surrounding microbiome. The CRISPR-Cas9 system can precisely alter plant DNA to achieve desired features, which can be used to produce rice with lower GHG emissions. In the quest to remove carbon, three steps can be defined for gene editing in rice crops. The first step is to strive to increase photosynthesis so that plants can absorb as much CO_2_ as possible, for example, by improving *OsHXK1* through the CRISPR-Cas9 system (Zheng et al., 2021). Second, efforts should focus on developing rice with longer roots and compatibility with lower GHG emissions. Rice plants transport carbon to the soil through their roots (as well as from crop residues upon harvest). Longer roots can bury carbon deeper in the ground, thus reducing the likelihood that it is released into the atmosphere (Kirschbaum et al., 2021). Third, the ability of the soil to retain GHGs must be increased rather than removing or converting GHGs to less destructive forms such as CH_4_ to CO_2_, N_2_O to N_2_, and CO_2_ to bicarbonates. Carbon is often not retained in the soil for long. One potential outcome of CRISPR-Cas9 research is a product that could be added to the soil to nurture a soil microbiome that holds on to carbon for a longer period of time (Jansson et al., 2023).

### Rhizosphere engineering to reduce GHG emissions from rice paddies

Rhizosphere engineering, which focuses on strategically manipulating plant–microbe interactions in the root zone to achieve desired outcomes (Khatibi et al., 2024), offers a promising strategy for reducing GHG emissions from rice paddies. There are four key strategies for mitigating CH_4_ emissions through rhizosphere engineering: (1) optimizing carbon allocation, directing more carbon toward rice grains; (2) regulating root exudate composition to lessen the preference for rhizospheric methanogens; (3) increasing the abundance of rhizospheric and endospheric methanotrophs; and (4) modifying root architecture to enhance oxygen transport (Kwon et al., 2024). Evidence indicates that redirecting photosynthates to favor seeds over roots can reduce CH_4_ emissions and increase rice yields (Kwon et al., 2023). In addition, modulating the composition of root exudates to decrease glucose levels could reduce CH_4_ emissions by up to 50% (Luo et al., 2022). Rice genes that induce the growth and activity of beneficial microbes, such as CH_4_ consumers or methanotrophs, can also significantly reduce CH_4_ emissions. Furthermore, limiting the formation of aerenchyma potentially results in lower CH_4_ emissions and reduces oxygen release from roots. Systematic breeding of hybrid rice varieties with optimized carbon allocation, regulated root exudates, minimal aerenchyma formation, intense oxidation activity, and effective induction of methanotroph activity could collectively help to reduce CH_4_ emissions in rice fields (Supplemental Figure 1). Nevertheless, the complexity of plant–microbe interactions in the rhizosphere makes it difficult to predict the outcomes of rhizosphere engineering. At the same time, environmental factors such as soil type, water management, and climate can have a considerable effect on its success. Future directions in rhizosphere engineering should focus on better understanding the intricacies of plant–microbe interactions in the rhizosphere and their influence on CH_4_ emissions. Advances in microbial ecology, genomics, and biotechnology offer opportunities to fine-tune rhizosphere engineering strategies, specifically the use of gene-editing tools to precisely manipulate rice root traits to optimize microbial interactions and reduce CH_4_ emissions. In addition, the integration of rice rhizosphere engineering with sustainable agricultural practices such as AWD irrigation could enhance its effectiveness.

### Using machine learning to develop low-carbon practices for hybrid rice systems

**Figure 6:**
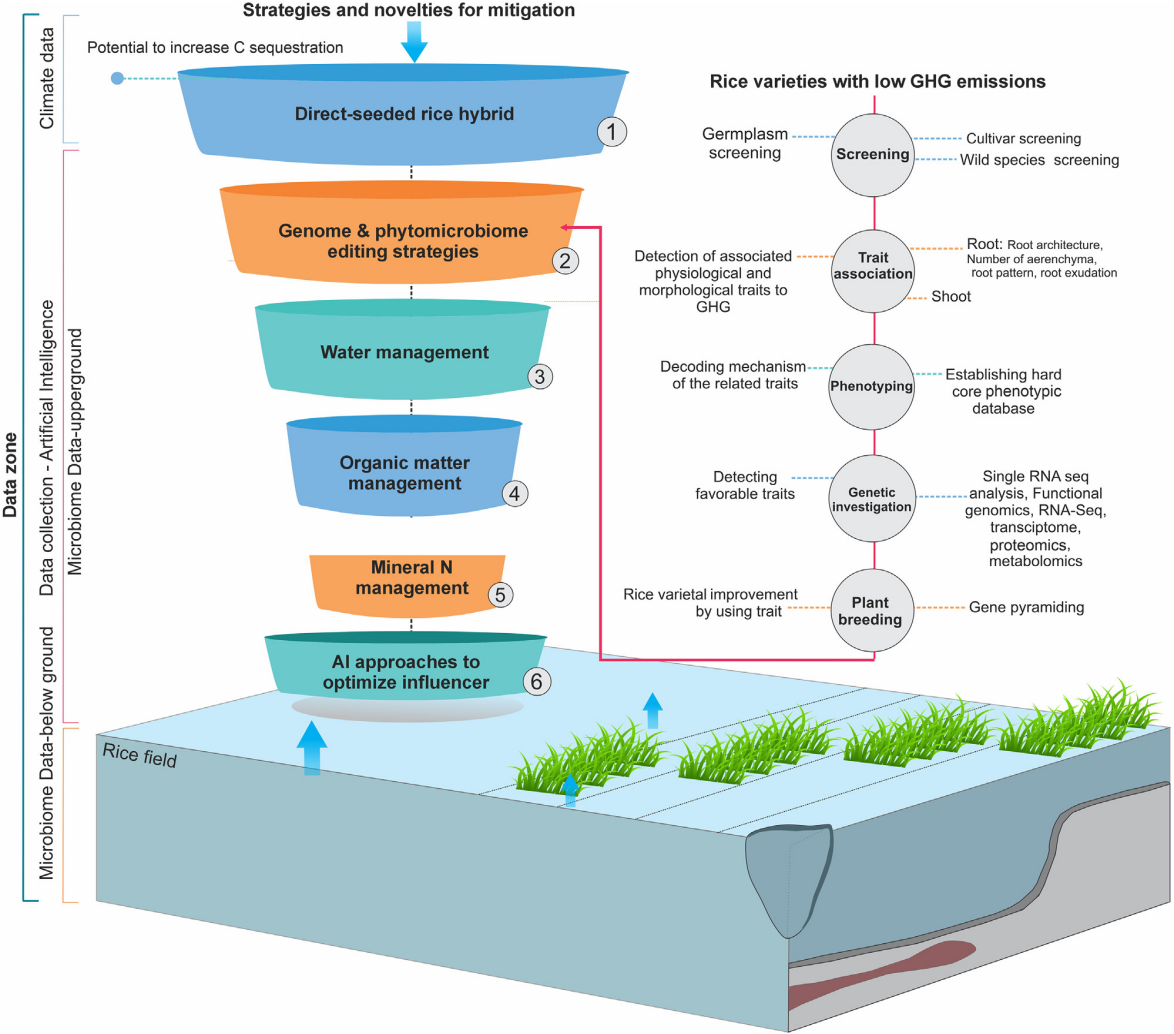
Strategic workflow and potential datasets for use in reducing the carbon footprint of rice paddies. The diagram explains the different types of datasets that could be used to extract comprehensive patterns for all major and minor drivers of GHG emissions from rice paddies. This uniform pattern can help develop the most feasible strategy to significantly reduce the carbon footprint of rice fields through robust approaches like AI and ML. AI and ML algorithms have a strong ability to understand complicated patterns that belong to different layers of GHG emissions from rice fields, such as omics, genetics, management practices, and environmental data.

To decipher the multi-layer complexity of GHG emissions and develop appropriate management practices for paddy fields, it will be necessary to consider all the drivers of GHG emissions in this complex environment. Machine learning (ML) and deep learning (DL) approaches can handle the complicated relationships between predictors and target variables and can therefore offer the advantages of rapid computation, good heterogeneity, and high prediction performance (Kamir et al., 2020; Khatibi and Ali, 2024). Recently, ML has been used to estimate GHG emissions from soil, primarily in drylands. For instance, a prior study used a random forest approach to estimate N_2_O emissions from no-tillage canola under various N application rates and found that moisture and soil N availability were the most crucial factors (Glenn et al., 2021). Another study evaluated the effectiveness of many ML models for forecasting soil CO_2_ and N_2_O emissions from oat, maize, and soybean rotation systems (Jiang et al., 2023). The results showed that the long short-term memory network model was more accurate than the root zone water quality model performance (Hamrani et al., 2020). ML has also been used to close the gaps in CH_4_ fluxes detected by eddy covariance (Irvin et al., 2021). Supplemental Table 2 presents the updated applications of ML and DL to CO_2_ and N_2_O emission research in the crop sector. However, plenty of opportunities remain to decipher the multi-layer complexity of GHG emissions from rice paddies using ML and DL approaches. The primary advantage of ML is its ability to enable classification, simplification, and forecasting with highly complex datasets, including diverse types of datasets with different types of data. The power of AI enables comprehensive monitoring and mobilization by analyzing data across larger geographic and temporal scales, enabling detailed observations of intricate processes. ML and DL can help to predict and select the best practices for management and comprehensive strategies applicable to all aspects of GHG challenges. Also, AI approaches have robust capacity and potential for decoding microbiome relations in rice plants with underlying agro/and molecular mechanisms with GHG emission from rice paddies. Since low-cost, high-throughput sequencing technologies have become available, the collection of microbiome data has become increasingly common. Because AI has massive potential to analyze highly complex data with multi-layer interactions, it may be the best option for automated decision-making when optimizing management systems and genomic selection to reduce CH_4_ and N_2_O emissions from rice fields. Figure 6 illustrates all the possible main drivers and parameters, together with accessible datasets, that should be considered for reducing GHG emissions from rice paddies. These multi-layer datasets could be used as model feeders for robust AI and ML models.

## Summary and future perspectives

Paddy rice is one of the most significant sources of CH_4_ and N_2_O emissions. The breakdown of organic matter produces CH_4_ under anaerobic conditions, whereas N_2_O is produced through nitrification and denitrification processes during the transition from flooded to dry conditions in rice fields. On the basis of *in situ* data, global CH_4_ emissions, N_2_O emissions, and yield-scaled GHG emissions from rice fields are estimated to average 283 kg CH_4_ ha^−1^, 1.7 kg N_2_O ha^−1^, and 0.9 kg CO_2_ kg^−1^, respectively. Under climate-change scenarios, these emissions are expected to rise and to reduce crop productivity. Field trials indicate that warming will result in a 15%–23% increase in worldwide CH_4_ emissions and a 10%–13% reduction in rice yield. Differences in CH_4_ and N_2_O emissions are affected mainly by irrigation, organic matter management, N fertilization, and rice selection. Understanding the major drivers of emissions and the processes that control their magnitude through system-level studies will likely improve the development of strategies for GHG mitigation. A key consideration for mitigation practices is that they must involve little to no modification of current crop management and farm equipment in order to increase grower adoption. Sustained high-yield performance with lower GHG emissions is a valuable metric and resource for rice breeders trying to address the negative effects of the climate crisis. Hybrid selection is an effective strategy that offers multiple benefits to farmers, such as sustained high grain yields, minimal yield-scaled GHG emissions, and tremendous potential for C storage in fields. Furthermore, the adoption of nutrient-use-efficient and direct-seeded rice hybrids will accelerate reductions in the C footprint of paddy fields.

Despite enormous amounts of research on GHG emissions and reduction approaches, the complex interactions among major drivers and how their effects are altered by different agronomic management practices remain unclear. Emerging techniques, such as ML and DL, have tremendous potential to bring about an understanding of the most sophisticated patterns and extract the best decisions using whole datasets collected on management practices, plant genotypes, phytomicrobiomes, and environmental and climate patterns. Advanced breeding techniques will also aid in the development of rice cultivars with lower GHG emissions under future climate-change scenarios. Genomic selection with high-throughput phenotyping, genome-wide association studies, ML/AI approaches, and genotyping strategies are important for the identification of genes for rice improvement to reduce GHG emissions. We must produce environmentally friendly genome-modified rice to combat these emissions using the CRISPR-Cas9 system or other new approaches. However, there are still several leading players that should be gaining attention to reduce GHG emissions; these include the phytomicrobiome, as one of the generators and regulators of GHG emissions in rice fields. Our current ability to disentangle the functional relationships between GHG emissions and soil microorganisms is made possible by advanced developments in molecular techniques for soil microbiology. This information could be instrumental in establishing novel and comprehensive mitigation strategies. In other words, to comprehensively understand the mechanisms and regulators involved in GHG emissions from rice fields, the best approach is to integrate all possible data from all main actors to extract a unique and realistic pattern, leading to the best and most optimized strategy for permanent reduction of the GHG emissions associated with rice production.

## Funding

The authors declare that financial support was received for the authorship and publication of this article. This publication was funded by the International Rice Research Institute (IRRI)–Hybrid Rice Development Consortium (HRDC) and the AGGRi Alliance project “Accelerated Genetic Gains in Rice Alliance” by the 10.13039/100000865Bill and Melinda Gates Foundation through grant OPP1194925-INV 008226.

## Acknowledgments

No conflict of interest is declared.

## Author contributions

S.M.H.K., M.A.A.-B., N.G.D., A.M.R., and J.A. contributed to the conceptualization, design, and manuscript writing; all authors contributed to revising the manuscript. All authors read and approved the manuscript.

## Supplemental information

Document S1. Supplemental Figure 1 and Supplemental Tables 1 and 2

Document S2. Article plus supplemental information
