# Supplementary material for: Advanced technologies for reducing greenhouse gas emissions from rice fields: Is hybrid rice the game changer?
Source: Plant Commun. 2024 Dec 28;6(2):101224. doi: 10.1016/j.xplc.2024.101224 (PMC11897460; doi:10.1016/j.xplc.2024.101224)
Supplement: Document S1. Supplemental Figure 1 and Supplemental Tables 1 and 2 [file mmc1.pdf]

Plant Communications, Volume *6*

Supplemental information

Advanced technologies for reducing greenhouse gas emissions from rice fields: Is hybrid rice the game changer?

Seyed Mahdi Hosseiniyan Khatibi, Maria Arlene Adviento-Borbe, Niña Gracel Dimaano, Ando M. Radanielson, and Jauhar Ali

Advanced technologies for reducing greenhouse gas emissions from rice fields: Is hybrid rice the game changer?

Seyed Mahdi Hosseiniyan Khatibi,[1] Maria Arlene Adviento-Borbe,[2] Niña Gracel Dimaano,[1,3] Ando M. Radanielson,[1] Jauhar Ali[1,*]

Affiliations/institutions

[1]International Rice Research Institute, Metro Manila, Philippines

[2]U.S. Department of Agriculture (USDA) Agricultural Research Service (ARS)

[3]College of Agriculture and Food Science, University of the Philippines Los Baños, Laguna, Philippines

***Corresponding author:**

Dr. Jauhar Ali

International Rice Research Institute, Metro Manila, Philippines

Email: J.Ali@irri.org

Short summary:

Rice contributes to 48% of GHG emissions from croplands. This review discusses opportunities for lowering GHG emissions and increasing crop productivity. Adoption of high-yielding and shorter duration rice hybrids that are identified as low-GHG emitters would significantly help combat the GHG emissions. Highlighted data driven approaches to understand and identify ways to reduce GHG emission from rice paddies.

This file includes:

Supplementary Table 1 and 2

Supplementary Figure 1

References

Supplemental Table 1. Key soil parameters influencing the output and emissions of N_2O and CH_4 from rice fields.

Parameter	Influence on CH_4 emission	Influence on N_2O emission
Moisture and aeration	Increased soil moisture results in a reduction in oxygen exchange, leading to anaerobic conditions in the soil respiration system that may impair the operation of methanotrophs and lead to methane emission.	When the soil moisture content in the paddy field is raised, the N_2O emission will decline. Oxidizing microbes are prevented from acting under a higher moisture levels.
Soil characteristics	More CH_4 was released from soil with a high organic carbon concentration than from clay soils with the same carbon content (Baggs et al., 2000). The influence of soil texture and mineralogy on soil puddling can further affect the rate of percolation in flooded rice fields and the net emission of methane. Non-clayey soils facilitate faster diffusion of soil gases due to less tortuosity and higher diffusivity rates (Kirk, 2004; Brye et al., 2013).	The rate of N_2O emission from soil increased due to a fine soil texture and without mobilization before sowing (Chen et al., 2008; Tan et al., 2009). This is due to the tiny aggregation of macropores developed in clayey soil, boosting anaerobic microsites and N_2O emissions. Sandy soil produced less N_2O emissions than clayey soils(Brentrup et al., 2000).
Soil temperature	The soil temperature significantly impacts methanogenesis processes, increasing or decreasing CH_4 generation. CH_4 formation begins at 15 to 20°C and will reach peak formation at about 37°C (Nozhevnikova et al., 2007).	An increase in the rate of N_2O emission results from increased soil respiration (microbial activity) in anaerobic areas (Akiyama et al., 2000; Signor and Cerri, 2013).
Soil pH	Most methanogens are neutrophilic because methane emission is more effective in the pH range between 6.5 and 7.5 (Jain et al., 2004). Methane release in the soil suspension is stopped by a pH range of 5.8 and 8.8.	While N_2O emission increased at low soil pH, the denitrification rate is slower in acidic soil than in slightly alkaline soil settings (Pathak, 1999). Therefore, the supply of nitrogen-based substrate for the synthesis of N_2O is reduced.
Soil chemical Condition	An appreciable amount of Mn^{2+}, NO_3^-, SO_4^{2-}, or Fe^{3+} mitigates production by preventing soil reduction (Achtnich et al., 1995).	NO_3− in soil may increase production by favoring denitrification, while others do not (Yagi et al., 1997).

Soil redox potential (Eh)	Production starts at − 150 to − 160 mV (Wang et al., 2017).	Production is considerably over +250 mV but not significantly below +200 mV (Wang *et al.*, 2017).

Supplemental Table 2. Recent research on prediction and measurement of CH_4 and N_2O based on machine learning approaches in crops.

Year	Machine learning application	Target	Reference
2024	Assessing methane emissions through environmental and UAV remote sensing variables	Rice paddy fields	(Velez et al., 2024)
2024	N_2O–N and CO_2–C emissions predictions	Decomposing rye cover crop	(Joshi et al., 2024)
2024	Geographical differences in the effect of biochar–A global simulation	Crop yield and greenhouse gas emissions	(Xu et al., 2024)
2024	Estimation and mitigation of nitric oxide emissions	Chinese vegetable fields	(Han et al., 2024)
2024	Automatic detection of methane emissions using a vision transformer	Multispectral satellite imagery	(Rouet-Leduc and Hulbert, 2024)
2024	Co-benefits for net carbon emissions through improved management of organic nitrogen and water	Rice field	(Khatibi and Ali, 2024; Liu et al., 2024)
2024	Optimizing agricultural management: A regional heterogeneity perspective	Soil greenhouse gas emissions and yield balance in China	(Li et al., 2024)
2023	Estimating energy consumption and GHG emissions: A machine learning approach	Crop production	(Sharafi et al., 2023)
2023	Modeling greenhouse gas emissions at different time scales	Irrigated paddy fields	(Jiang et al., 2023)
2023	Predicting Maize theoretical methane	Yield in combination with ground and UAV remote data	(Kavaliauskas et al., 2023)
2023	Quantitative assessment and mitigation strategies of greenhouse gas emissions	Rice fields in China	(Wu et al., 2023)
2023	Methane emissions across Monsoon Asia	Paddy rice	(Ouyang et al., 2023)

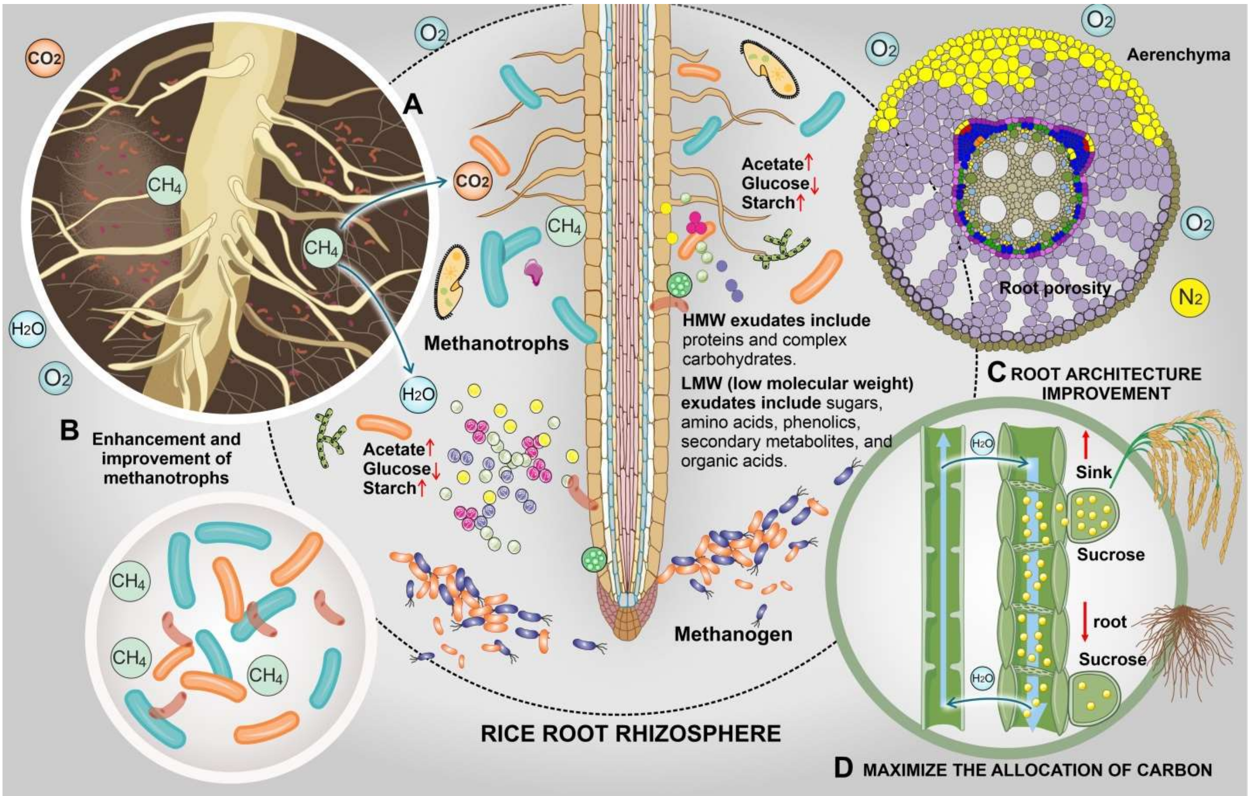

Supplemental figure 1. Engineering rice rhizosphere and traits to reduce methane emission.

(A) Rice rhizosphere and its interactions/cross-talking with the microbiome

(B) Increase the abundance of methanotrophs in the rice endospheric and rhizosphere regions.

(C) Root architecture improvement to transport more oxygen.

(D) Optimize photosynthate allocation to the root and sink organs.

References

Achtnich, C., Bak, F., and Conrad, R. (1995). Competition for electron donors among nitrate reducers, ferric iron reducers, sulfate reducers, and methanogens in anoxic paddy soil. Biology and fertility of soils **19**:65-72. https://doi.org/10.1007/BF00336349

Akiyama, H., Tsuruta, H., and Watanabe, T. (2000). N2O and NO emissions from soils after the application of different chemical fertilizers. Chemosphere-Global Change Science **2**:313-320. https://doi.org/10.1016/S1465-9972(00)00010-6

Baggs, E., Rees, R.M., Smith, K., and Vinten, A. (2000). Nitrous oxide emission from soils after incorporating crop residues. Soil use and management **16**:82-87. https://doi.org/10.1111/j.1475-2743.2000.tb00179.x

Brentrup, F., Küsters, J., Lammel, J., and Kuhlmann, H. (2000). Methods to estimate on-field nitrogen emissions from crop production as an input to LCA studies in the agricultural sector. The international journal of life cycle assessment **5**:349-357. https://doi.org/10.1007/BF02978670

Chen, S., Huang, Y., and Zou, J. (2008). Relationship between nitrous oxide emission and winter wheat production. Biology and Fertility of Soils **44**:985-989. https://doi.org/10.1007/s00374-008-0284-4

Han, Z., Leng, Y., Sun, Z., Lin, H., Wang, J., and Zou, J. (2024). Machine learning-based estimation and mitigation of nitric oxide emissions from Chinese vegetable fields. Environmental Pollution **343**:123174. https://doi.org/10.1016/j.envpol.2023.123174

Jain, N., Pathak, H., Mitra, S., and Bhatia, A. (2004). Emission of methane from rice fields-A review.

Jiang, Z., Yang, S., Smith, P., and Pang, Q. (2023). Ensemble machine learning for modeling greenhouse gas emissions at different time scales from irrigated paddy fields. Field Crops Research **292**:108821. https://doi.org/10.1016/j.fcr.2023.108821

Joshi, D.R., Clay, D.E., Clay, S.A., Moriles-Miller, J., Daigh, A.L., Reicks, G., and Westhoff, S. (2024). Quantification and machine learning based N2O–N and CO2–C emissions predictions from a decomposing rye cover crop. Agronomy Journal **116**:795-809. https://doi.org/10.1002/agj2.21185

Kavaliauskas, A., Žydelis, R., Castaldi, F., Auškalnienė, O., and Povilaitis, V. (2023). Predicting Maize Theoretical Methane Yield in Combination with Ground and UAV Remote Data Using Machine Learning. Plants **12**:1823. https://doi.org/10.3390/plants12091823

Khatibi, S.M.H., and Ali, J. (2024). Harnessing the power of machine learning for crop improvement and sustainable production. Frontiers in Plant Science **15**:1417912. https://doi.org/10.3389/fpls.2024.1417912

Li, H., Jin, X., Shan, W., Han, B., Zhou, Y., and Tittonell, P. (2024). Optimizing agricultural management in China for soil greenhouse gas emissions and yield balance: A regional heterogeneity perspective. Journal of Cleaner Production **452**:142255. https://doi.org/10.1016/j.jclepro.2024.142255

Liu, B., Guo, C., Xu, J., Zhao, Q., Chadwick, D., Gao, X., Zhou, F., Lakshmanan, P., Wang, X., and Guan, X. (2024). Co-benefits for net carbon emissions and rice yields through improved management of organic nitrogen and water. Nature Food:1-10. https://doi.org/10.1038/s43016-024-00940-z

Nozhevnikova, A.N., Nekrasova, V., Ammann, A., Zehnder, A.J., Wehrli, B., and Holliger, C. (2007). Influence of temperature and high acetate concentrations on methanogenensis in lake sediment slurries. FEMS microbiology ecology **62**:336-344. https://doi.org/10.1111/j.1574-6941.2007.00389.x

Ouyang, Z., Jackson, R.B., McNicol, G., Fluet-Chouinard, E., Runkle, B.R., Papale, D., Knox, S.H., Cooley, S., Delwiche, K.B., and Feron, S. (2023). Paddy rice methane emissions across Monsoon Asia. Remote Sensing of Environment **284**:113335. https://doi.org/10.1016/j.rse.2022.113335

Pathak, H. (1999). Emissions of nitrous oxide from soil. Current science:359-369.

Rouet-Leduc, B., and Hulbert, C. (2024). Automatic detection of methane emissions in multispectral satellite imagery using a vision transformer. Nature Communications **15**:3801. https://doi.org/10.1038/s41467-024-47754-y

Sharafi, S., Kazemi, A., and Amiri, Z. (2023). Estimating energy consumption and GHG emissions in crop production: A machine learning approach. Journal of Cleaner Production **408**:137242. https://doi.org/10.1016/j.jclepro.2023.137242

Signor, D., and Cerri, C.E.P. (2013). Nitrous oxide emissions in agricultural soils: a review. Pesquisa Agropecuária Tropical **43**:322-338. https://doi.org/10.1590/S1983-40632013000300014

Tan, I.Y., van Es, H.M., Duxbury, J.M., Melkonian, J.J., Schindelbeck, R.R., Geohring, L.D., Hively, W.D., and Moebius, B.N. (2009). Single-event nitrous oxide losses under maize production as affected by soil

type, tillage, rotation, and fertilization. Soil and Tillage Research **102**:19-26. https://doi.org/10.1016/j.still.2008.06.005

Velez, A.F., Alvarez, C.I., Navarro, F., Guzman, D., Bohorquez, M.P., Selvaraj, M.G., and Ishitani, M. (2024). Assessing methane emissions from paddy fields through environmental and UAV remote sensing variables. Environmental Monitoring and Assessment **196**:574. https://doi.org/10.21203/rs.3.rs-3909062/v1

Wang, C., Lai, D.Y., Sardans, J., Wang, W., Zeng, C., and Peñuelas, J. (2017). Factors related with CH4 and N2O emissions from a paddy field: clues for management implications. PloS one **12**:e0169254. https://doi.org/10.1371/journal.pone.0169254

Wu, Q., Wang, J., He, Y., Liu, Y., and Jiang, Q. (2023). Quantitative assessment and mitigation strategies of greenhouse gas emissions from rice fields in China: A data-driven approach based on machine learning and statistical modeling. Computers and Electronics in Agriculture **210**:107929. https://doi.org/10.1016/j.compag.2023.107929

Xu, X., Li, T., Cheng, K., Yue, Q., and Pan, G. (2024). Geographical differences in the effect of biochar on crop yield and greenhouse gas emissions–A global simulation based on a machine learning model. Current Research in Environmental Sustainability **7**:100239. https://doi.org/10.1016/j.crsust.2023.100239

Yagi, K., Tsuruta, H., and Minami, K. (1997). Possible options for mitigating methane emission from rice cultivation. Nutrient Cycling in Agroecosystems **49**:213-220.
